# Supplementary figures and images for: Type I interferons and MAVS signaling are necessary for tissue resident memory CD8+ T cell responses to RSV infection
Source: PLoS Pathog. 2022 Feb 2;18(2):e1010272. doi: 10.1371/journal.ppat.1010272 (PMC8843175; doi:10.1371/journal.ppat.1010272)

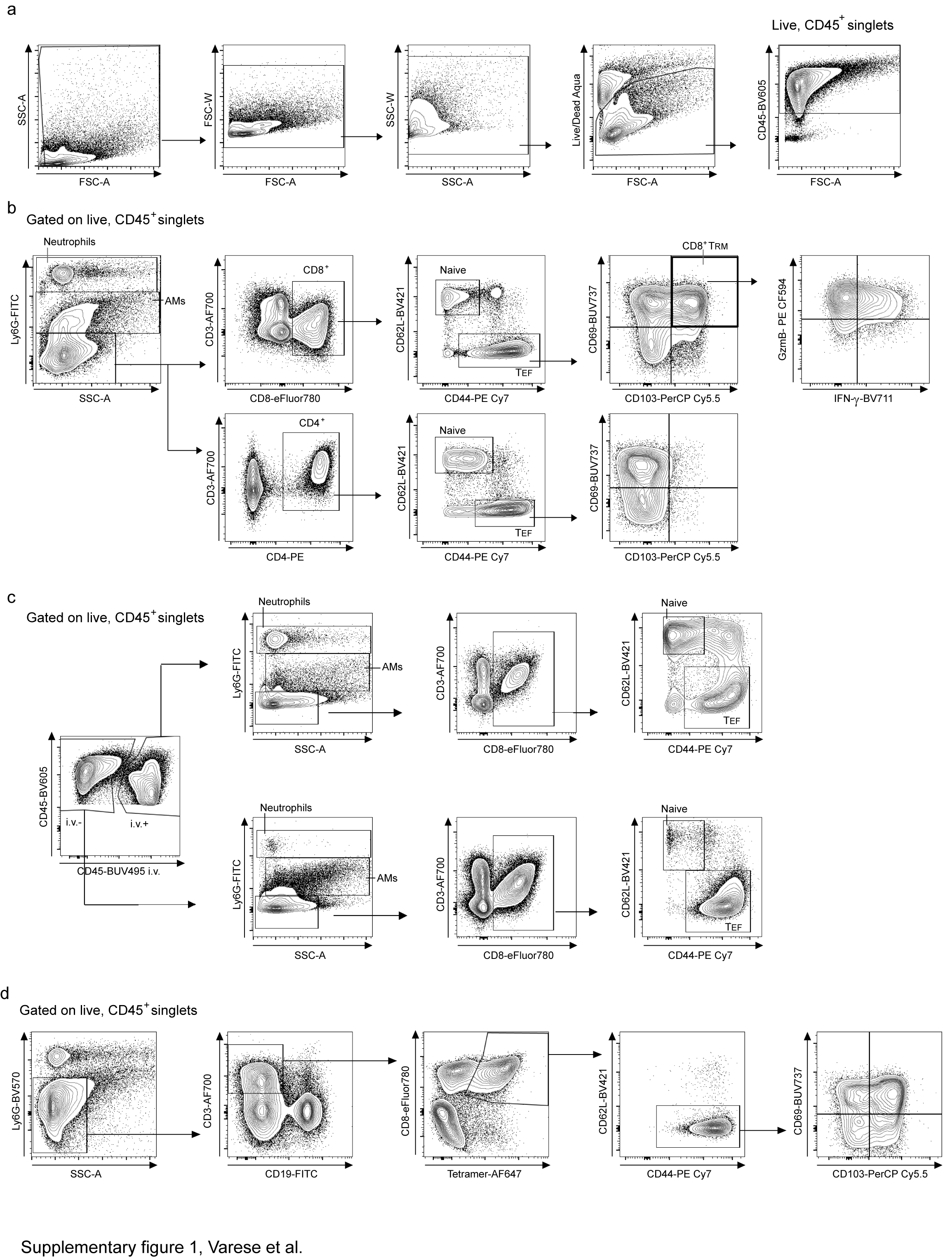

Supplement: S1 Fig — Lung cells were obtained after collagenase digestion and BAL cells obtained and stained for different surface and intracellular markers. Representative plots from lung cells are shown. (a) Flow cytometry analysis was performed on 250,000 CD45+ events after excluding debris, doublets and dead cells. (b) Gating strategy used to identify neutrophils, alveolar macrophages (AMs), naïve CD8+ and CD4+ T cells, CD8+ and CD4+ TEF cells, CD8+ TRM cells and IFN-γ and GzmB producing CD8+ TRM cells. (c) Strategy used for identification of vasculature (i.v.+) or lung resident (i.v.-) leukocytes using i.v. in vivo staining with anti-CD45 BUV394. (d) Gating strategy used to identify M187-195 specific CD8+ T cells. All gates were defined using fluorescence minus one (FMO) controls for each antibody used. The same gating strategy was used in airway cells purified from bronchoalveolar washes. (TIF) [file ppat.1010272.s001.tif]

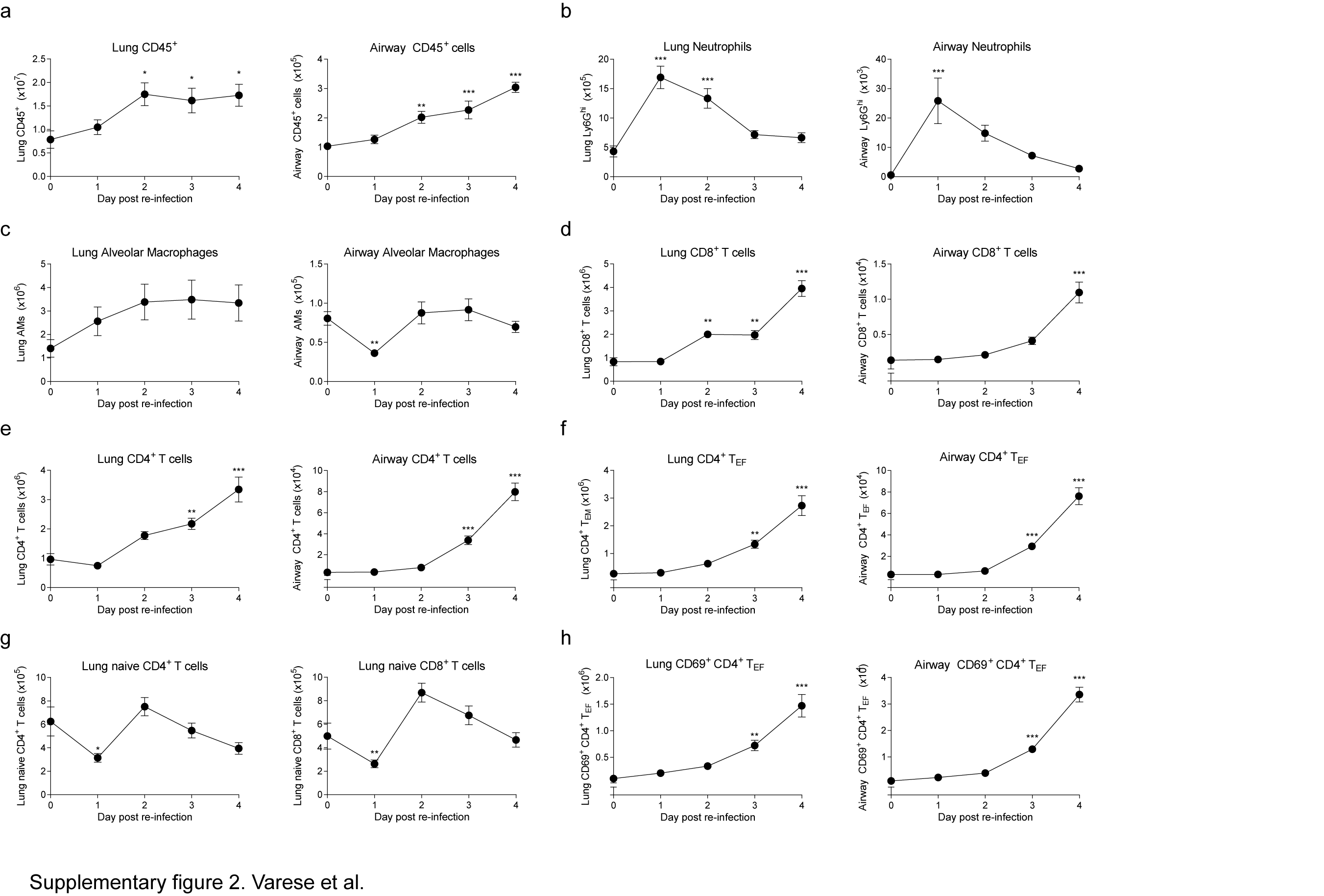

Supplement: S2 Fig — Mice were RSV infected i.n.. At 3 weeks p.i. mice were mock or re-challenged with RSV (day 0 = mock re-infection) and lung and airway cells were analyzed at the indicated time-points by flow cytometry. Total lung and airway (a) CD45+ cells, (b) neutrophils, (c) alveolar macrophages (AMs), (d) total CD8+ T cells, (e) total CD4+ T cells, (f) effector (CD62L- CD44+) CD4+ T cells, (g) lung CD8+ and CD4+ naïve T cells, and (h) lung and airway CD69+ CD4+ effector (CD62L- CD44+) T cells were quantified by flow cytometry. Data are presented as the mean ±SEM of 9–11 individual mice per time point, pooled from two independent experiments. Statistical significance of differences between day 0 (mock re-infected) and other time points was determined by one-way ANOVA with Tukey’s post hoc test. * indicates differences between day 0 and days 1–4. * P ≤ 0.05, ** P ≤ 0.01, *** P ≤ 0.001. (TIF) [file ppat.1010272.s002.tif]

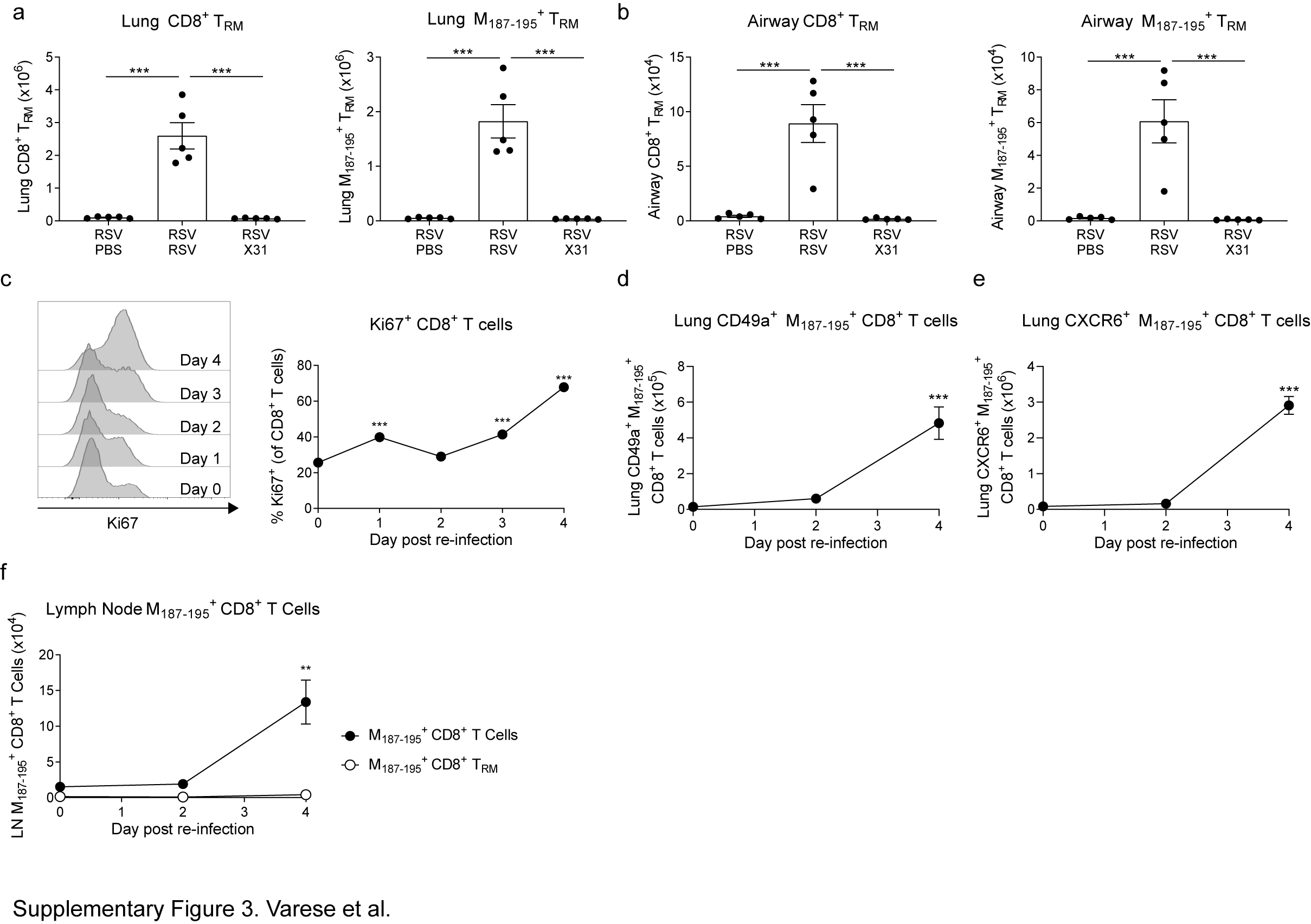

Supplement: S3 Fig — RSV infected mice were intranasally re-infected with RSV or infected with influenza A virus (X31 strain; 250 PFU per mice). Four days later mice were euthanized and (a) TRM cells and (b) M187-165 specific TRM cells were quantified in lungs and BAL. (c) Representative histograms showing Ki67 expression on CD8+ T cells in lung at different days post RSV re-infection. Vasculature CD45 cells were labeled in vivo by intravenous injection of anti-CD45 antibody and i.v. CD45- (d) CD49a+ and (e) CXCR6+ M187-195+ cells were quantified in lungs at different days post re-infection. (f) i.v. CD45- M187-195 specific CD8+ T cells and TRM cells were quantified in lung draining lymph nodes (LN) after RSV re-infection. Panels a, b, c show data of one representative experiment. In panels d, e and f data are presented as the mean ±SEM of 9–11 individual mice per time point, pooled from two independent experiments. Statistical significance was determined by one-way ANOVA with Tukey’s post hoc test. In panels d, e, f * indicates differences between day 0 and days 1–4. * P ≤ 0.05, ** P ≤ 0.01, *** P ≤ 0.001. (TIF) [file ppat.1010272.s003.tif]

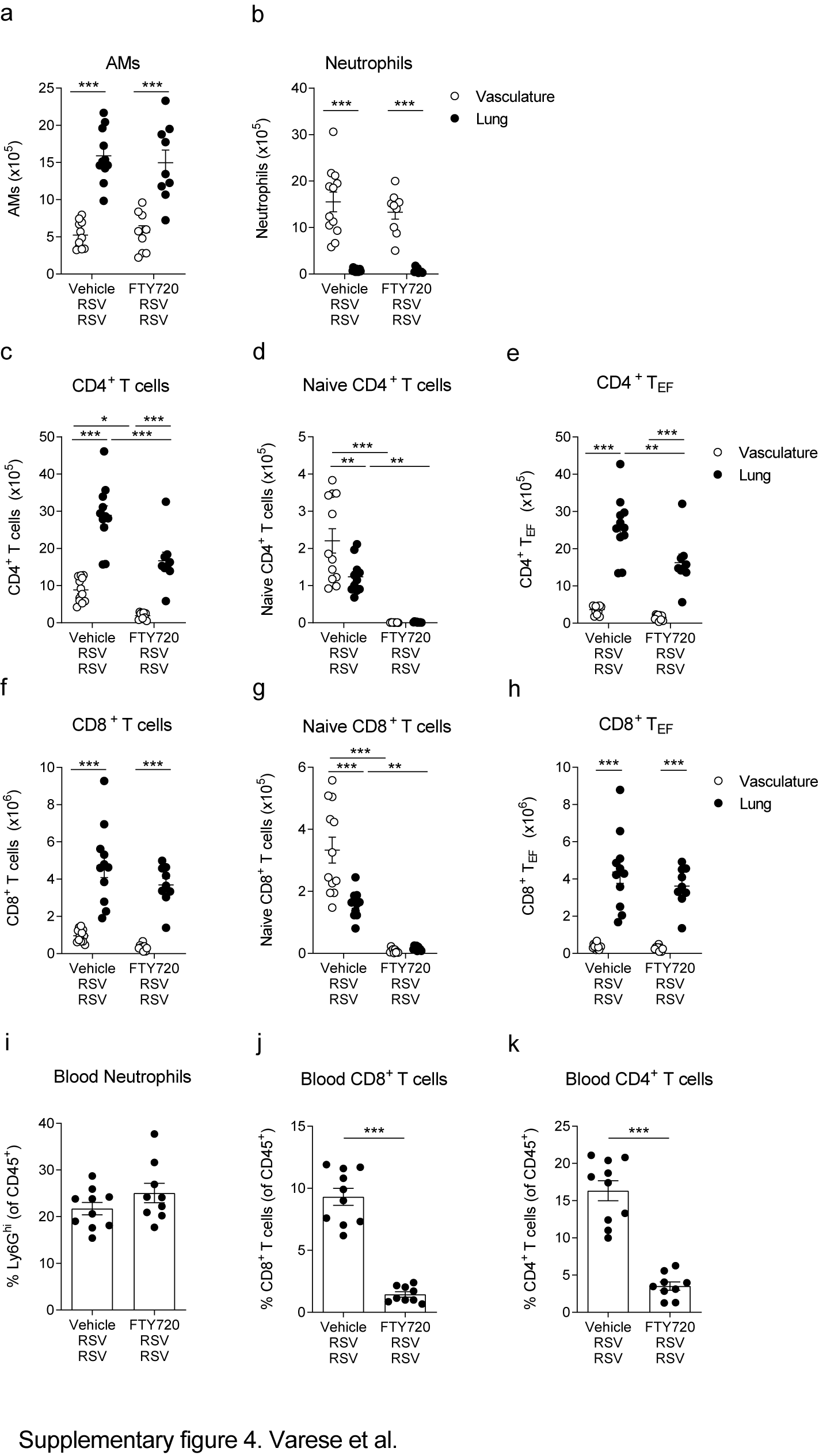

Supplement: S4 Fig — Mice re-infected with RSV were treated with 25 μg FTY720 i.p. administered daily from day -2 prior to re-challenge until day 3 post RSV re-challenge. Mice were given 2μg of CD45-BUV395 i.v. 10 min prior to euthanasia to distinguish cells in the vasculature of the lung from resident lung cells. (a) Alveolar macrophages (AMs), (b) neutrophils, (c) total CD4+, (d) naïve CD4+ and (e) effector (CD62L- CD44+) CD4+ T cell, (f) total CD8+, (g) naïve CD8+, and (h) effector (CD62L- CD44+) CD8+ T cell were assessed by flow cytometry discriminating between vascular (i.v. CD45+) and resident (i.v. CD45-) populations. Blood samples were obtained immediately post-mortem from the femoral vein and (i) neutrophil, (j) CD8+ and (k) CD4+ T cells were quantified by flow cytometry. Data are presented as the mean±SEM of 7 PBS re-infected (RSV/PBS), 12 vehicle-treated and 9 FTY720-treated re-infected individual mice pooled from two independent experiment. Statistical significance of differences between groups was determined by one-way ANOVA with Tukey’s post hoc test. * P ≤ 0.05, ** P ≤ 0.01, *** P ≤ 0.001. (TIF) [file ppat.1010272.s004.tif]

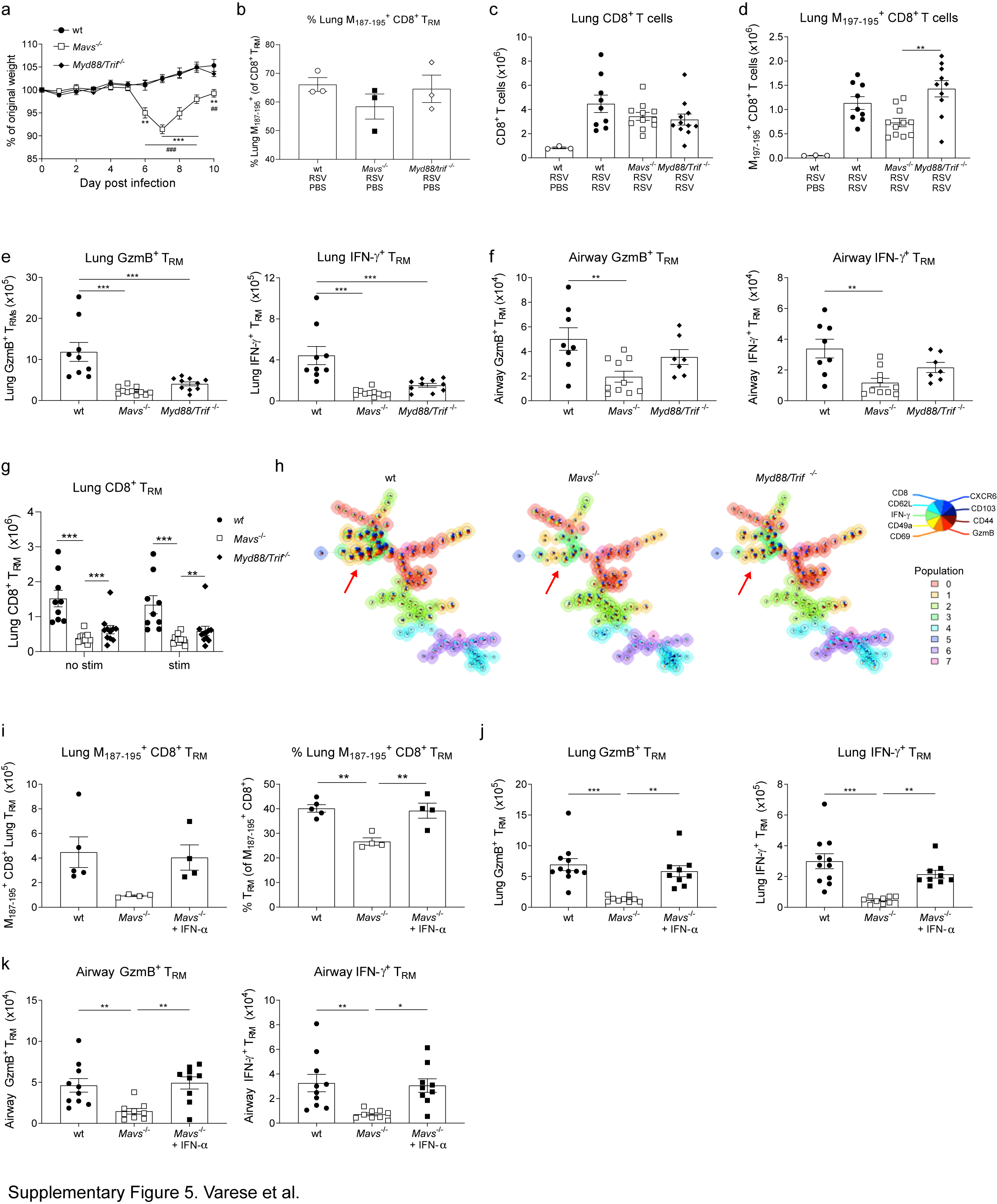

Supplement: S5 Fig — (a) wt, Mavs-/- and Myd88/Trif -/- mice were infected i.n. with RSV. Body weight was monitored throughout primary infection and percentage of original weight was quantified. (b) Lung cells were recovered stained with tetramers loaded with M187-195 RSV peptide to quantify the percentage of RSV-specific cells within the CD8+ TRM cells after primary infection. Mice were re-infected with RSV and 4 days later(c) total and (d) RSV-specific CD8+ T cells were quantified. Lung and airway cells were stimulated with RSV M187-195 peptide and IFN-γ and GzmB production was detected by intracellular staining and quantified in CD8+ TEM cells (CD62- CD44+) cells using flow cytometry. Total number of IFN-γ and GzmB positive CD8+ CD69+ CD103+ TRM cells in (e) lung tissue and (f) airways. (g) Number of TRM cells was quantified in the absence and presence of M187-195 peptide stimulation (h) RSV M187-195 peptide stimulated CD8+ T cells were analyzed using dimensionality reduction software (UMAP) and automatic cluster identification (FlowSOM). FlowSOM tree-plots for each genotype are presented, showing pie charts with marker expression for each node and color identification of each of the 8 identified populations (corresponding to populations shown in Fig 5G). Pie chart diameter represents the proportion of each node in the data set. Red arrow indicates major change between wt, Mavs-/- and Myd88/Trif -/- mice. Mavs-/- mice were treated i.n. at 6h and 18h during primary RSV infection with 500ng of recombinant IFN-α. Four days after re-infection mice were euthanized and (i) M187-195 specific TRM cells were quantified in the lungs, and GzmB+ and IFN-γ+ TRM cells were quantified in ex vivo M187-195 peptide stimulated (j) lung and (k) BAL cells. In a, data are presented as the mean±SEM of 19 wt, 19 Mavs-/- and 19 Myd88/Trif -/- mice pooled from three independent experiment. Statistical significance of differences between groups was determined by two-way ANOVA with Tukey’s post hoc test. In b-d [file ppat.1010272.s005.tif]

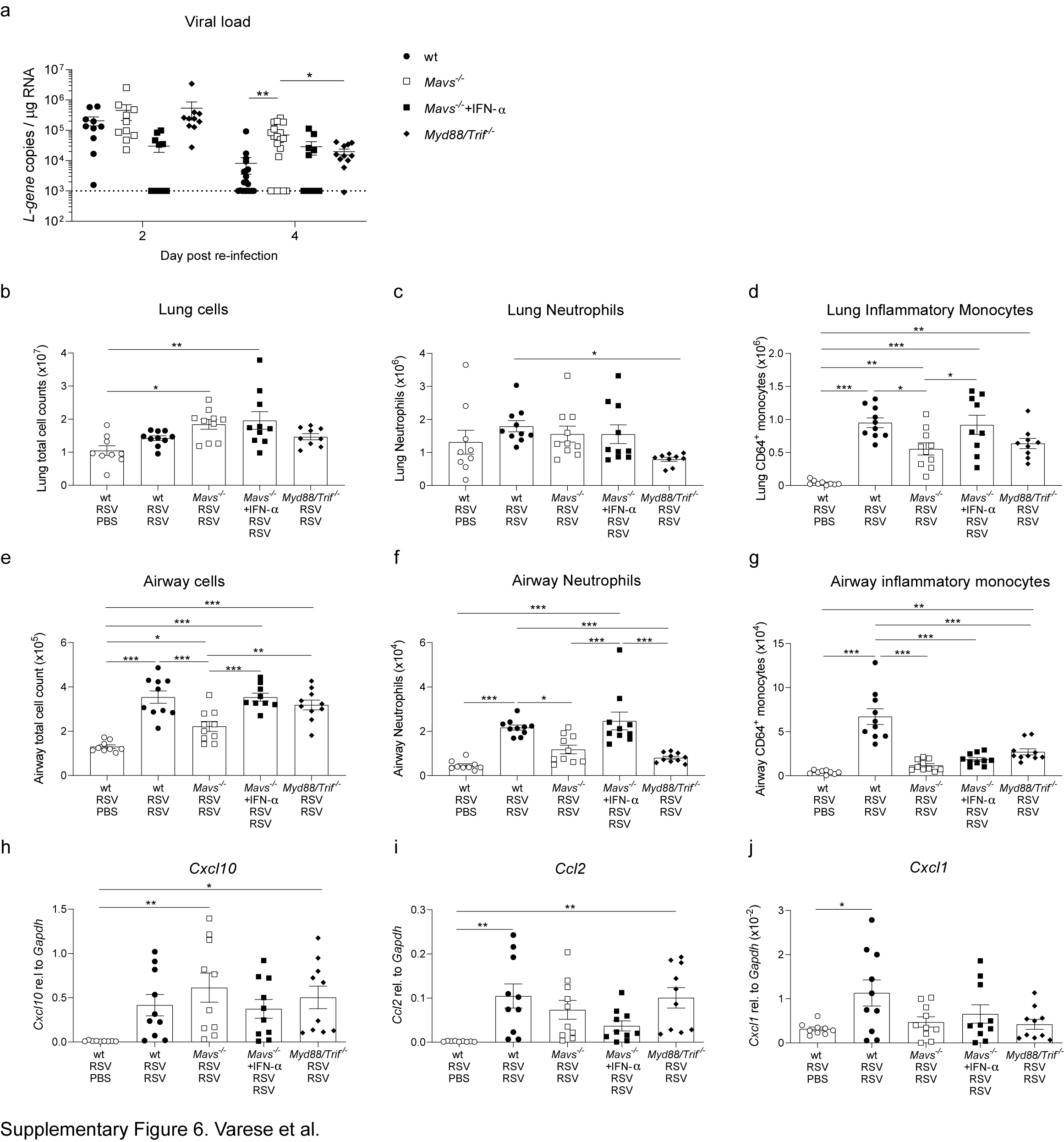

Supplement: S6 Fig — RSV infected wt, Mavs-/-, Myd88/Trif -/- mice and IFN-α treated Mavs-/- mice were re-infected with RSV and euthanized at day 2 and 4 post re-infection. (a) Viral load was determined in lung tissue by L-gene detection by qPCR. Lung cells were recovered and (b) total cell count, (c) neutrophils and (d) CD64+ inflammatory monocytes were quantified in the lung by flow cytometry. BAL cells were recovered and (e) total cell count, (f) neutrophils and (g) CD64+ inflammatory monocytes were quantified in the lung by flow cytometry. Cxcl10, Ccl2 and Cxcl1 expression levels in lung tissue were determined by qPCR. Data are presented as the mean±SEM of 9–11 mice per group pooled from two independent experiment. Statistical significance of differences between groups was determined by one-way ANOVA with Tukey’s post hoc test. * indicates differences between groups. * P ≤ 0.05, ** P ≤ 0.01, *** P ≤ 0.001. (TIF) [file ppat.1010272.s006.tif]
