# Supplementary material for: Type I interferons and MAVS signaling are necessary for tissue resident memory CD8+ T cell responses to RSV infection
Source: PLoS Pathog. 2022 Feb 2;18(2):e1010272. doi: 10.1371/journal.ppat.1010272 (PMC8843175; doi:10.1371/journal.ppat.1010272)
Supplement: Attachment — Submitted filename: Rebuttal to PLOS Pathogens final.docx [file ppat.1010272.s008.docx]

**Rebuttal to PLOS Pathogens**

**Manuscript ID D-21-01420R1 "Type I interferons and MAVS signalling are necessary for tissue resident memory CD8^+^ T cell responses to RSV infection”**

We thank the reviewers and editor for very constructive feedback and we detail our changes and additions to the manuscript below.

**Overview:**

*Reviewer #1: The manuscript by Varese et al., explores the role of innate immune components in the generation and re-call of lung Trm following RSV infection. They show that mice lacking key components of innate signalling pathways (MAVS) display an impaired local expansion and activation of lung Trm following RSV challenge. Interestingly, the introduction of type I interferon in MAVS mice during a primary RSV infection resulted in the development of lung Trm with improved capacity to undergo secondary recall expansion although these cells still retained defects in recall effector function. This is an interesting and well written study supported by robust data. However, one major caveat present throughout the paper is that in most experiment profiled bulk CD8+ memory T cells instead of RSV specific cells. It does appear the group has access to RSV tetramers and therefore I strongly recommend that they go back and repeat some of the key experiments tracking the virus specific cells which would provide more informative information than profiling the bulk memory T cell pool.*

We thank the reviewer for his/her support and for the constructive feedback. As the reviewer suggested, we have repeated some of the experiments to include quantification of RSV-specific T_RM_ cells by tetramer staining. Please find more details in the comments below.

*Reviewer #2: This manuscript examines the role of type I IFNs and MAVS signaling in the generation and effector functions of tissue resident memory CD8 T cells specific to respiratory syncytial virus (RSV) in mice. The authors perform initial kinetics of the Trm response after RSV re-infection including an examination of granzyme B expression and IFN-gamma production capacity by the cells following peptide restimulation. Additional experiments examining the response in MAVS or Myd88/Trif deficient mice suggest that MAVS plays an important role in the expansion and effector functions of the Trm following RSV reinfection. Additional experiments indicate tat type IFNs during the primary RSV infection may be critical to the initial generation of the Trm cells in the lung. The manuscript is well written and the experiments are generally well designed. However, a major limitation of the manuscript is the failure to use the widely utilized in vivo labeling approach to distinguish cells within the lung tissue from those in the vascular space which is likely to impact the interpretation of a number of the critical experiments performed at day 4 after virus rechallenge when there will be many cells trafficking towards the lung in the blood that may not be in the lung tissue at that timepoint.*

We again thank the reviewer for his/her support and for useful suggestions/feedback. We had included one experiment using the in vivo labelling technique in the first submission. We agree with the reviewer that it is important to show this more extensively and have now included more experiments where we can distinguish the truly resident T_RM_ cells from the one in the vascular compartment (please see additional comments below).

*Reviewer #3: In this manuscript the studies have addressed the presence of Trm cells with clearance of RSV upon reinfection. These data are important since many studies have identified Trm cells as critical for the long term protection of re-infection. Trm cells are most often examined within the tissue and are present upon the reinfection to quickly respond to the pathogen locally. These studies have further suggested that MAVS mediated activation is important for the generation of Trm cells upon reinfection. The studies also suggested that the presence of type I IFN during the primary infection was critical for reconstituting the "protective" reinfection response. These conclusions are logical and follow previous data from this and other groups.*

We thank the reviewer for the for his/her support and have included more specific responses to the feedback below.

**Major Issues: Key Experiments Required for Acceptance**

**Reviewer #1:**

*1. Fig 1a-f –The analysis of Trm numbers following re-infection should be restricted to antigen specific tetramer+ cells.*

We have now extended our results by using tetramer staining and/or intracellular staining for granzyme B and IFN-γ after RSV peptide stimulation as readouts for RSV specific T_RM_ cells. We show tetramer^+^ CD8^+^ T cells in new Fig. 1h and j, Fig. 4e-h, Supp. Fig. 3a, b, d-f, Supp. Fig. 5b, d, h and i and granzyme B and IFN-γ production after RSV peptide stimulation in Fig. 2e-g, Fig. 5a-c, Fig. 6g-i, Supp. Fig. 5e, f, j and k.

*2. Fig 1d – It would be informative if the authors could compare the numbers of effector T cells seen following reinfection to that seen during a primary infection.*

We have characterised the response to primary infection in previous work (Paulsen, M. et al. Frontiers in immunology **11**, 52020 and Goritzka, M. et al. The Journal of Experimental Medicine **212**, 699–714, 2015). We now explicitly mention that the numbers of total CD4^+^ and CD8^+^ T cells at day 4 of re-infection is similar to the number of cells at the peak of the primary response. Please see lines 128-130 in the Results section.

*3. Fig 1e - It has been previously documented by others that Trm undergo local recall expansion in tissues following reinfection. Can the authors show whether there is also evidence of expansion in the lung draining LN. It would be interesting to see whether there is a difference in kinetics of recall at local (lung) and distal (LN) sites.*

We thank the reviewer for this comment. We had not analysed the lymph nodes and, therefore, set up new experiments to address this point. Our new data from two new independent experiments (included in Supp. Fig. 3f) show that there is a modest expansion (compared to lung) of RSV-specific CD8^+^ T cells in the lymph node after RSV re-infection but no expansion of T_RM_ cells (i.e. CD103^+^ CD69^+^).

*4. Fig 1a-d - Is the expansion of CD8 T cells in the lung following secondary infection with RSV antigen specific – would infection with a different respiratory pathogen (ie influenza) also see rapid increase in Trm numbers?*

This is another useful suggestion by the reviewer. To address this point, RSV-infected mice were infected three weeks later with influenza A virus (X31) and CD8^+^ T cells and T_RM_ cells were quantified. These data are shown in Supp. Fig. 3a-b and indicate that there is no expansion of T_RM_ cells 4 days post influenza A virus infection of previously RSV infected mice. In other words, the expansion of T_RM_ cells upon RSV re-infection is antigen-specific.

*5. Supplementary Fig 2 –It would be informative to compare the kinetics of immune cell infiltration into the lung following secondary infection to that observed during primary infection.*

We have previously characterised the kinetics of immune cell infiltration during primary infection (Goritzka, M. et al. The Journal of Experimental Medicine 212, 699–714, 2015 and Kirsebom, F. C. M., et al. Mucosal Immunology 12, 1244–1255, 2019). We now explicitly mention that the kinetics of the innate immune cells during secondary infection are similar although the magnitude of the response much lower than what is detected during primary infection (see lines 125-126). We have also included new data for immune cell infiltration at day 2 post re-infection in wildtype, *Mavs*^-/-^ and *Myd88/Trif*^-/-^ mice (Supp. Fig. 6b-g) and we discuss the similarities and differences in the response to the re-infection compared to primary infection (see lines 217 to 222).

6*. Fig 2a - Is the rapid clearance of RSV following secondary exposure mediated by the local T cells. Would CD8 or CD4 T cell depletion prior to secondary infection impact viral control? Also, it would be informative to compare these titres to those seen after a primary infection.*

In the RSV model, mice develop good and long-lasting antibody responses. As it is not possible to do a heterologous infection with RSV, the secondary infection will not be as extensive as the primary infection as the virus is cleared very quickly. It is therefore likely that a direct effect on the viral load by T_RM_ cells will be difficult to detect in this model. Nevertheless, to address the reviewer’s comment, we depleted CD8^+^ cells before re-infection and quantified viral load at day 2 post infection (the peak of virus titer after re-infection shown in Fig. 2). Even though the depletion of CD8^+^ T cells was efficient, we did not detect a statistically significant difference in viral load (see figures below). Therefore, this model does not allow us to use reduction in viral load as a readout of T_RM_ cell function.

Figure for reviewers. CD8^+^ cells were depleted just before and during RSV re-infection. Although CD8^+^ T cells were depleted in the lung, there was no effect on viral load at day 2 post re-infection.

*7. Fig 2e-g – The authors are using CD69+CD103 expression to identify Trm in these ex vivo peptide stimulation assays. Does peptide stimulation ex vivo impact the expression of these markers (and therefore affect the interpretation of this assay). Can the authors show the frequency of CD69+CD103+ T cells in these assays with and without peptide stimulation – if peptide stimulation is not altering expression, these should match.*

We thank the reviewer for pointing this out and have compared the T_RM_ cells in assays with or without peptide stimulation. We detected no difference in the expression levels of CD69 and CD103 confirming that we can compare the data from the different readouts. We have added these data to Supp. Fig. 5g.

*8. Fig 3 – It would be more informative if tetramer+ cells were tracked in this experiment.*

We agree with the reviewer that tracking RSV-specific T_RM_ cells is important and have now included more data on tetramer positive cells Fig. 1, 4, Supp. Fig 3, and Supp. Fig. 5. As all CD8^+^ T_RM_ cells are found in the lung after FTY720 treatment, this would include both RSV tetramer^+^, other RSV-specific and, possibly, other CD8^+^ T_RM_ cells. The main point of this figure is to show that the CD8^+^ T_RM_ cells are all lung resident. From an ethical and cost perspective, we therefore did not repeat these experiments to include tetramer staining.

*9. Fig 4c – Can the authors show fold change in Trm numbers post re-infection in WT, MAVS-/- and Myd88/Trif KO. It is hard to compare defects in expansion when there are differences in starting numbers of Trm. Once again – it would be useful to show tetramer+ cells rather than bulk CD8 T cells in this experiment.*

We have also evaluated the tetramer^+^ CD8^+^ T_RM_ cells at baseline and show that equal frequencies of RSV-specific T_RM_ cells are found in the wildtype, *Mavs*^-/-^ and *Myd88/Trif^-^*^/-^ mice (Supp. Fig. 5b). However, we agree with the reviewer and now also indicate the fold increase in T_RM_ cells from baseline (prior to re-infection) to day 4 post re-infection. We have added these data to the text (see lines 226-228).

*10. Fig 4i – was UMAP analysis performed on tetramer+ cells? If not, it would interesting to compare the profile presented to that of the tetramer+ cells.*

Yes, the UMAP was performed on tetramer^+^ cells, which is now more clearly highlighted in the text, figure and figure legend.

*11. To show that the impaired Trm pool generated following primary infection of MAVS-/- mice has a biological impact on the capacity to control a secondary infection viral loads in the lung of reinfected wt and MAVS-/- mice should be compared. Moreover, it would be interesting to see whether IFNa treatment of MAVS mice during the primary infection impacts the capacity of the animals to control a secondary infection. Do viral loads in the lung following re-infection of these mice differ from MAVS-/- not subjected to IFNa treatment?*

As the re-infection model of RSV does not allow for substantial viral replication (see reply to comment 6), detecting viral load as a determinant of the biological impact of T_RM_ cells is not possible. However, to see if we could detect any differences, we measured viral load at day 2 and 4 in wildtype, *Mavs*^-/-^, IFN-α treated *Mavs*^-/-^ and *Myd88/Trif*^-/-^ mice (Supp. Fig. 6a). There were no significant differences between the groups at day 2. On day 4, a time point when most of the virus has been cleared, there was a difference between wildtype mice and *Mavs*^-/-^ mice (please see more comments on Reviewer 3 point 2). However, there was no difference detected between wildtype and IFN-α treated *Mavs*^-/-^ mice. We discuss this in the manuscript (please see lines 323-329 in the discussion section).

**Reviewer #2:**

*1. A major limitation of the study is that the authors have not used in vivo antibody labeling to distinguish cells in the lung tissue from the peripheral blood. This is a major issue when performing these types of studies as many memory cells with similar phenotypes can be found in the blood and not present in the lung tissue. This is a major concern particularly when examining the memory response in RSV immune mice re-challenged with RSV and looking at day 4 as at this time point many cells will be trafficking towards the lung and it is important to distinguish the cells in the lung tissue from those in the peripheral blood to properly interpret the data and fully support the conclusions the authors attempt to make from their results. Perfusion of the lung is not adequate to remove the cells that will be in the small capillary beds of the lung which can result in an artificially high estimate of the cells in the lung tissue.*

We had some experiments in which we had used *in vivo* antibody labelling to distinguish resident from vascular T cells but have now extended these data (now shown in Fig. 1h-j, Fig. 3, Supp. Fig 3d-f, Supp. Fig 4). These new data indicate that the contribution of memory cells coming in from the blood is limited and does not impact the interpretation of our study. We thank the reviewer for encouraging us to more clearly make the point that the cells we are studying are extravascular.

*2. Total numbers of cells should be reported in Figure 5b-c in addition to the frequencies as in the other figures. This is a critical omission necessary to interpret the data presented and support the conclusions of this key point in the paper.*

*3. Similar to point 2 above for Figure 6h-i.*

The reviewer is correct and we usually show both frequencies and numbers of cell types. For the granzyme B and IFN-γ production, frequency highlights the proportion of CD8^+^ T_RM_ cells that is RSV-specific. However, we have now also added numbers of granzyme B and IFN-γ producing cells (Supp. Fig. 5e-f and j-k). We have also highlighted this in the text (please see lines 298-300). We thank the reviewer for suggesting that we include such data.

**Reviewer #3:**

*1. The concept of Trm cells is that they are resident in the tissue prior to a re-infection challenge and protect the host from the response. While the cell surface phenotype of the Trm cells were examined, they were primarily detected after 4 days of re-infection. Can these truly be considered Trm cells? How long after 4 days do these cells persist in the lung? By definition these cells exist in tissue and do not recirculate.*

The reviewer makes several important points. We used the drug FTY720 in Figure 3 to confirm that the T cells we are studying are not part of the recirculating pool. We also use *in vivo* labelling of cells in the vasculature to distinguish tissue resident cells from cells that are in the lung vasculature (more data on this added in the revised manuscript Fig 1h-j). Based on the reviewer’s comment, we were interested to find out how long the resident T_RM_ cells are present and have now analysed them at 2 and 4 weeks post infection. These data are now included in Fig 1i and j and show that RSV-specific CD8^+^ T_RM_ cells remain detectable in the lungs long after infection.

*2. The primary endpoint that is examined, especially in the Mavs deficient animal studies is weight loss. The studies should examine additional endpoints, including viral clearance and inflammation/histopathology. The weight loss measurement has been primarily shown to be accompanied by TNF and other innate cytokines. What is the cellular infiltration in the lungs at 8 days post-infection when the peak pathology (i.e. weight loss) occurs? Are there increased/decreased numbers of granulocytes, eosinophils and neutrophils?*

The reviewer is correct and the weight loss in evident during primary infection. We have previously studied the viral load and cellular infiltration during primary infection (Goritzka, M. et al. The Journal of experimental medicine **212**, 699–714, 2015 and Kirsebom, F. C. M., et al. Mucosal Immunology **12**, 1244–1255, 2019). Following the suggestion from the reviewer, we focused on determining viral load and inflammatory responses after re-infection in wildtype mice and *Mavs*^-/-^ mice. There was no difference in viral load at day 2 post infection but a difference is seen at day 4 (now shown in Supp. Fig. 6a), a timepoint when most virus has been cleared in wildtype mice but the *Mavs^-^*^/-^ mice are lagging behind. We discuss this in the manuscript (please see lines 213-217 and 323-329 in the discussion section). For the inflammatory response, we evaluated chemokines and cell infiltration post re-infection in *Mavs*^-/-^, IFN-α treated *Mavs*^-/-^ and *Myd88/Trif*^-/-^ mice (now shown in Supp. Fig. 6b-j). Overall, we found in secondary infection a similar profile of chemokines and cell types infiltrating the lung as we detect during primary infection but the magnitude of the response is much smaller. This has been discussed on lines 217-224).

*3. An additional parameter that might be helpful in these studies would be lung physiology. Are there functional consequences to the differences in responses within the different groups at anytime during the re-infection?*

Unfortunately, we do not have access to a lung function apparatus in our animal facility. However, we do not detect any weight loss or other signs of disease during RSV re-infection. In that setting, it is unlikely that we would see an effect of re-infection on airway resistance and any differences among groups.

*4. While the data clearly demonstrates that there are secondary infection consequences that are occurring in the Mavs deficient mice, the characterization of what are the responses in the primary responses with or without type I IFN would be important. What are the changes in the primary immune response when Mavs is absent and when type I IFN is reconstituted?*

The reviewer is correct as there are differences in the response to RSV in *Mavs^-^*^/-^ mice. We have previously extensively characterised the RSV response in *Mavs*^-/-^ mice (Goritzka, M. et al. The Journal of experimental medicine **212**, 699–714, 2015 and Kirsebom, F. C. M., et al. Mucosal Immunology **12**, 1244–1255, 2019). We now discussed this in more detail in the revised manuscript as those data provide the background to IFN-α treatment of *Mavs*^-/-^ mice. Please see lines 348-358 in the discussion section.

**Part III – Minor Issues: Editorial and Data Presentation Modifications**

**Reviewer #1: (No Response)**

**Reviewer #2:**

*1. It is unclear why there is twice as many IFN-g producing CD8 Trm cells between Fig 2 E at day 4 and Fig 5a at day 4 which should essentially be the identical conditions.*

These experiments have been done at different times. We have noticed a variation in IFN-γ staining between experiments, but we always detect the same differences in the IFN-γ frequencies between groups.

*2. Examination of total GzmB protein levels and Ifng RNA levels are not very informative since it is much more important to know the changes in both the frequency and total number of cells capable of making these molecules.*

Yes, we agree with the reviewer but still consider the overall response as an informative readout. However, we have added all the frequencies and numbers of cells to Supp. Fig. 5.

**Reviewer #3: (No Response)**
